# Defining chronic pain impact levels: a patient-clinician approach using PROMIS® pain interference scores

**DOI:** 10.1186/s41687-025-00908-y

**Published:** 2025-08-18

**Authors:** Karon F. Cook, Dokyoung Sophia You, Michael Von Korff, Sean C. Mackey

**Affiliations:** 1 Feral Scholars, 257 County Road 4754, Broaddus, 7592 TX USA; 2 Department of Family and Community Medicine, University of Oklahoma-Tulsa, Tulsa, OK USA; 3 Kaiser Permanente Washington Health Research Institute (Retired), Seattle, Washington USA; 4 Department Anesthesiology, Perioperative and Pain medicine, Stanford University School of Medicine, 1070 Arastradero Road,Suite 200, MC 5596, Palo Alto, CA 94304 USA

**Keywords:** Pain, High impact chronic pain, Interpretation of HRQOL data, Item response theory (IRT), Psychometric methods/scaling

## Abstract

Our study aims to establish PROMIS Pain Interference score (PI) ranges that clinicians and persons living with chronic pain (PLwCP) associate with none, mild, moderate, and high impact chronic pain. We employed the PRO-Bookmarking technique to identify threshold scores that delineate different levels of chronic pain impact. PROMIS-PI score vignettes or “score stories” were developed to communicate the experience of living with different levels of pain interference. A panel of 10 PLwCP and another of 5 pain clinicians identified pairs of score vignettes they judged to represent the threshold between two levels of pain impact (e.g., ‘‘moderate impact” and ‘‘high impact”). We defined threshold scores as the mean score of the adjacent vignettes. We applied the obtained threshold scores to the distribution of PROMIS-PI scores in a sample of persons (n = 31,090) seen in a tertiary pain clinic. In another sample, we compared the Bookmarking and the revised Graded Chronic Pain Scale (GCPS-R) classifications. Patients and clinicians were in consensus on threshold scores for “no impact” to “mild impact” and “moderate impact” to “high impact,” scores of 47 and 69, respectively. However, for the threshold for “mild impact to “moderate impact,” the value was 65 for patients and 60 for clinicians. The comparison of classifications in the secondary sample revealed differences, especially for the 3rd level, high impact. The GCPS-R classified 58.2% as having high impact chronic pain; the Bookmarking thresholds classified 23.8% as such. The PRO-Bookmarking approach effectively delineated thresholds for classifying levels of chronic pain impact using PROMIS-PI scores. This method incorporates the perspectives of PLwCP and allows for post-hoc application to diverse patient samples.

**Supplementary Information:**

The online version contains supplementary material available at 10.1186/s41687-025-00908-y.

## Introduction

Chronic pain affects 50–100 million U.S. adults, with an economic burden amounting to $500 billion annually [1, 2]. Among this chronic pain population, approximately 20 million Americans experience “high impact chronic pain” (HICP) [3], facing significant challenges and having the highest needs [4]. The importance of HICP is highlighted by the goal to “decrease the prevalence of HICP” within the Healthy People 2030 initiative [5, 6]. To alleviate HICP burden, the first step is to clearly define and validly operationalize the concept. A robust classification system for different pain impact levels will undergird the development of effective treatments for HICP.

The National Pain Strategy defines HICP as pain occurring on at least half of the days ≥ 6 months and imposing substantial restriction in work, social, and self-care activities [7]. Despite its face validity, this definition poses significant measurement challenges. The Graded Chronic Pain Scale-Revised (GCPS-R) is widely used to classify pain impact levels [4] and, to date, it is the only measure for doing so. The GCPS-R defines HICP as pain that limits “life or work activities” most or every day, over the last 3 months [4]. Specific “life and work activities” subdomains are not defined, leaving the respondent to decide which activities to reference and how to weigh their impact.

Alternative measures, often labeled as “pain interference,” exist. NIH’s *Patient-Reported Outcomes Measurement Information System-*Pain Interference (PROMIS®-PI) [8] assesses all levels of pain impact and all subdomains such as pain impact on work, cognition, emotional function, recreation, sleep, and social function. A recent qualitative study shows that the PROMIS-PI items align well with patient-reported pain impact [9], supporting the content validity for its use in discriminating levels of chronic pain impact. PROMIS-PI measures are scored on a T-score metric (M = 50, SD = 10), with a wide range of achievable scores. For example, T-scores on the PROMIS-PI 8a short form range from 40.7 to 77.0. Compared to the GCPS-R, PROMIS-PI scores offer finer discrimination of pain impact levels, but this advantage comes with a practical barrier. The GCPS-R yields 4 discreet scores that operationalize 4 distinct levels of pain impact. There is no equivalent system for classifying respondents’ levels of pain impact based on PROMIS-PI scores.

The Bookmarking method [10], commonly used to set educational standards, has been adapted to define levels of patient-reported outcomes (PROs) [11]. This method relies on the expertise of people with chronic pain, clinicians, researchers, and other stakeholders to set meaningful thresholds, frequently called “cut points.” These cut points carve the pain interference metric into ranges of scores that stakeholders identify as representing different levels of a symptom or outcome. Each score range is identified based on a “plain language” label that stakeholders recognize and intuitively understand (e.g., none, mild, severe). This enhances the interpretability of PRO scores.

Our study aims to establish threshold scores using PRO-Bookmarking to differentiate pain impact levels based on PROMIS-PI scores, incorporating input from patients and clinicians. After defining these thresholds, we further characterize the distribution of pain impact in a large sample of patients from a tertiary pain clinic. We also compare these distributions using the newly defined bookmarking-based cut points on PROMIS-PI with the GCPS-R.

## Methods

The Stanford Institutional Review Board approved all study procedures. Informed consent was obtained from all participants.

### Measures

A demographic survey was administered to collect sex, age, race and ethnicity, education, marital status, employment, and disability status of participants.

#### PROMIS pain interference measure

The PROMIS-PI measure assesses multiple subdomains of pain interference. Items are scored 1–5; each response includes a verbal descriptor [8, 12], PROMIS-PI measures are calibrated on a T-score metric (M = 50, SD = 10) and centered on a large sample representing the 2000 US General Census demographics. Bookmarking Panel members completed the PROMIS-PI Short-Form v1.1 8a. Responses were scored using a web-based scoring system (http://www.healthmeasures.net/). Higher PROMIS-PI T-scores indicate greater pain interference. Two comparison datasets were used to compare Bookmarking results—one from a previous research study and the other from a large clinic sample. For the former, we used the PROMIS-PI v1.1 8a short form; for the latter, PROMIS-PI was administered using computer adaptive testing (CAT; T-scores ranged from 38.6 to 83.8).

#### The graded chronic pain scale-revised (GCPS-R)

Both PROMIS-PI and the 6-item GCPS-R were administered in a previous study and the results were used to interpret Bookmarking findings. The GCPS-R assesses the degree of pain impact (mild, bothersome/moderate, high impact chronic pain) [4]. Respondents report how often they have had pain (first item) and life or work limiting pain (second item) in the last 3 months (never, some days, most days, every day). Patients who respond most days or every day to both items are categorized as having HICP. The other levels of chronic pain are identified based on summing 3 items scaled 0–10 that ask about pain intensity, pain interference with enjoyment of life, and interference with general activities (PEG score). The PEG score of ≥ 12 will be classified into the bothersome/moderate and < 12 will be classified into the mild chronic pain impact. A graphic explaining the scoring rubric of the GCPS-R is available elsewhere [4].

### Samples

#### Bookmarking sample

##### Patient panelists

 We aimed to recruit 10 to 13 people living with chronic pain (PLwCP) to comprise a panel that would elicit opinions about levels of pain impact using the Bookmarking method. Participants were recruited using our learning healthcare system (CHOIR; https://choir.stanford.edu/), a platform that collects and integrates patient-reported outcomes into clinical care and research to support data-driven and patient-centered care, and real-world discovery [13]. We sent an email invitation with a prescreening survey link to patients who had previously agreed to receive information about research opportunities. Inclusion criteria were being at least 18 years of age and reporting chronic musculoskeletal pain. Because the informed consent, study materials, and procedures were conducted solely in English, participants with no or limited English proficiency were excluded. Interested patients completed a prescreening survey to assess pain conditions, English fluency, and demographic information. The demographic information allowed us to invite diverse participants to comprise the Bookmark panel. Specifically, our recruitment goal was to include at least one male, one female, one older adult (aged 65 or older), and one participant with a high school education or less. The prescreening survey asked whether the potential participant could attend a 4-hour, in-person session on a Saturday or Sunday in July 2021. After setting a date for the Bookmarking event, a research coordinator contacted eligible patients to explain the study. Interested participants were emailed the informed consent form. We paid patient participants $75 for participating in this study.

##### Physician panelists

Physician panelists were recruited by email invitation to all pain physicians practicing at the Stanford Pain Management clinic. Those who were interested and available on the date of the Bookmarking event participated in this study. Physician participants were paid $400.

#### Comparison samples for assessing consequential validity

In the context of PRO Bookmarking, “consequential validity” is supported if the results of an assessment and its classification rubric are logically consistent with the intended use. We applied the thresholds derived in this study to two comparison samples and evaluated the results.

##### Learning health care sample

Using CHOIR, our learning healthcare system, we routinely administer patient-reported outcome measures during initial and subsequent medical visits to assess relevant risk factors, guide treatment decisions, and evaluate treatment outcomes. We extracted the PROMIS-PI T-scores of 31,090 patients seeking care at a tertiary pain center. For the current study, we only extracted the initial survey data. This sample was used to create a frequency distribution of levels of chronic pain impact based on the Bookmarking results.

##### Social determinants of health study sample

In prior work, we conducted a study investigating social determinants of health in an inclusive sample of patients with mixed chronic pain etiologies and anatomical locations. Participants completed both the GCPS-R and the PROMIS-PI Short Form 8a, allowing us to compare differences in chronic pain impact based on the GCPS-R rubric and the Bookmarking-based thresholds. Participants for this study were recruited at a tertiary pain clinic (n = 409) and received a $15 Amazon gift card for their participation. They had previously agreed to be contacted about research opportunities.

### Procedures

A total of 4,370 patients who had previously agreed to receive information about research were sent notifications of the study. During the first day, 657 completed the prescreening survey (15% response rate). Because we deemed this a sufficient participant pool from which to obtain the 10–13 participants needed, the invitation link was discontinued after only one day. A research coordinator phoned 112 participants from this pool to confirm their eligibility and their availability for an in-person PRO Bookmarking activity. Selection of participants to invite was based on their availability for a 4-hour in-person visit on a specific date and our recruitment targets (i.e., at least one in each of these categories: male, female, age ≥ 65, high school education or less). Screening concluded after 13 patients agreed to participate.

#### Development of score vignettes

For each item of the PROMIS-PI item bank, and for each possible score, we calculated the most probable response. For example, for the item, “How much did pain interfere with your household chores?” the most likely responses for scores of 50, 60, and 70 are “not at all”, “somewhat”, and “very much”, respectively. We used an R-program that, based on a measure’s IRT-calibrated item parameters, outputs the most likely responses for each item across all possible scores (available from the authors). For the Bookmarking activity, we wanted to represent the entire range of PROMIS-PI scores with vignettes spanning the full score continuum. Two of the authors (Cook and You) selected potential items for vignettes with close attention to content balancing; that is, items were chosen from among several subdomains of pain interference (e.g., activities of daily living, cognitive impact, social impact, etc.). Eight vignettes were required to represent the full range of possible PROMIS-PI scores. As in previous studies, each vignette was assigned a personal surname to assist participants in referring to them (e.g., Ms. Smith).

In previous work, we noticed that, though each item of a vignette was chosen based on being the most likely response for a given T-score, the set of items may not exactly represent that target score. This is because a given response is the most likely one for a range of scores, not a single score. Responses to a set of items, however, are much more precise. To account for this, we scored each vignette as though it was an individual’s responses to the items of a custom short form. This was accomplished using the HealthMeasures.net Scoring Service [14]. Scored vignettes are more accurate, but they have the disadvantage of not landing on the targeted increments (e.g., 5 T-score points). The final set of 8 vignettes, their calibrated scores, and their names are presented in the Appendix. For the current study the threshold values were 47, 55, 60, 65, 69, and 76.

#### Bookmarking procedures

##### Panel of PLwCP

A facilitator (Cook) and supporting staff gathered into a room along with the PLwCP who comprised the Bookmarking panel. The facilitator gave a presentation that described the research study and how the results would be used. In addition, participants completed the PROMIS-PI short form version 8a.

Next, Panelists participated in a warm-up exercise to familiarize them with Bookmarking. Details of this exercise have been published [11]. This activity followed the procedures for Bookmarking pain interference vignettes, but, instead of score vignettes, several types of desserts were presented in order from least to most “fancy”. Participants defined levels of fanciness by placing bookmarkers for 3 levels of “fanciness” (i.e., plain/fancy/extra fancy). The use of this warm-up activity is commonly used in PRO Bookmarking.

The facilitator then sought participant input on names for levels of chronic pain impact. We started with the question, “Suppose for a moment that your clinician asked you how much pain impacted you last week. What words might you use to describe your pain impact?” As needed, the facilitator provided examples of levels used to distinguish gradations of other PRO outcomes. Once the group agreed on the names for the different levels, the facilitator led a discussion of what distinguished each level from the others, taking brief notes on a whiteboard. During this time study staff hand-wrote the agreed upon names for level of chronic pain impact onto paper bookmarks for participants use during the Bookmarking activity.

Next study staff handed out the prepared bookmarks and copies of the 8 vignettes we developed to represent the range of possible PROMIS-PI scores. The vignettes were numbered 1–8 according to their score (least to most chronic pain impact). Panel members were seated at tables and directed to lay the papers in numerical order before them. The facilitator explained that each of these was a story about how hypothetical patients answered items about pain’s impact on their lives over the past 7 days. Next, panelists were directed to read each vignette starting with “Ms. Thomas,” the vignette representing the least pain impact. Working independently, participants placed the first bookmark in front of the first person whose impact would be better described by the second level of impact (e.g., mild impact) than by the lowest level (e.g., no impact). In placing their bookmarks, panelists were told that 1) they did not have to have an equal number of people in each level, and 2) there were no wrong answers. Participants continued until they had placed all their bookmarks. They then recorded their bookmark locations on a recording sheet that was collected by the study team.

Panel members were told that 100% agreement on the placement of bookmarks was not required, but that we hoped to reach a consensus. Participants were asked to listen respectfully to everyone’s reasons for a bookmark’s placement and to be open to changing it if the discussion persuaded them. All panelists shared their initial placements for the first bookmarker by a show of hands, and the number of people selecting each location was recorded on a whiteboard. The facilitator invited people with different placements to explain how they chose their bookmark location. When there was disagreement, the facilitator asked participants to describe how they reached their decisions. As needed, the facilitator referred to the whiteboard, directing panelists’ attention to what they had previously said were distinguishing features of levels of chronic pain impact. Participants were asked to report if they wanted to change their bookmark location. Discussion continued until everyone was comfortable with their bookmark placements. This process was repeated for remaining bookmarks. Once all bookmark placements were decided, participants recorded their final bookmarks on a recording sheet.

##### Panel of pain physicians

For the physician panel, all procedures were the same as those for the PLwCP panel, with one exception. The physician panel was not asked to generate labels for levels of chronic impact. They used the labels generated by the PLwCP panel.

#### Analyses

Analyses were descriptive. Threshold values were calculated as the mean score of the two vignettes adjacent to each bookmarker. For example, a bookmark placement between adjacent vignettes scored at 63 and 66 would be a threshold score of 65. The distributions of individual participants’ final threshold values were calculated and displayed graphically. Modal values for each threshold were taken as the group thresholds, calculated separated for the PLwCP and physician panels.

The modal threshold scores were applied to two comparison samples to evaluate the consequential validity of the study results. Though we found no previous studies that estimated the incidence of HICP among patients seeking health care in a tertiary pain clinic, we expected the percentage to be high. This expectation was tested by applying the impact threshold scores obtained in this study in such a sample. Though we did not expect the GCPS-R classifications to align exactly with the PROMIS-PI classifications, we did expect that there would be similarity in the results. We tested this by applying the GCPS-R and PROMIS-PI for Social Determinants of Health Study participants. Results in both comparison samples were displayed graphically.

## Results

### Panel participant characteristics

Demographic characteristics are summarized in Table 1. As the table shows, we met our recruitment targets with the inclusion of 1 panelist with a high school diploma or GED, 3 males, and a mixture of participants who were Asian (n = 2), White (n = 6), and Other (n = 2). Panelists also varied by marital and employment and disability status. Of the 5 physician participants, 1 endorsed Asian and 4 endorsed White. The mean years in practicing pain medicine was 8 years (range 1–23 years).

**Table 1:** Participant demographics

	Panel of PLwCP (n = 10)		Panel of Pain Physicians (n = 5)		Learning Health Care Sample (n = 31,090)		Social Determinants of Health Study Sample (n = 409)		
	Number	%	Number	%	Number	%	Number		%
Sex									
Female	7	70	2	40	20,306	65	293		72
Male	3	30	3	60	9,922	32	105		26
Other or missing					802	3	11		3
Mean age (SD), range	58 (13),	38–78	46 (13),	34–59	52 (17),	18–104	56 (16),		18–91
Race									
White/Caucasian	6	60	4	80	16,535	53	325		79
Asian	2	20	1	20	2,824	9	25		6
Black American	0	0			1,027	3	16		4
Other*	2	20			10,704	34	43		11
Ethnicity									
Non-Hispanic	6	60			22,195	71	370		90
Hispanic	3	30			3,140	10	28		7
Other**	1	10			5,755	19	11		3
Education									
< high school	0	0			1,367	4	2		< 1
High school/GED	1	10			2,195	7	9		2
Some college	3	30			3,839	12	113		28
College degree	4	40			7,620	25	115		28
Advance degrees	2	20			6,530	21	162		40
Other***					9,539	31	8		2
Marital status									
Married/Partnered	7	70					239	58	
Divorced/widowed	1	10					93	23	
Never Married	2	20					72	18	
Other***	0	0					5	1	
Employment status									
Full-time	3	30					97	24	
Part-time	1	10					51	12	
Student	1	10					11	3	
Not working	1	10					102	25	
Retired	4	40					139	34	
Other***	0	0					9	2	
Disability Status									
No	7	70					293	72	
Yes	3	30					116	28	

Other* includes American Indian or Alaska Native, Pacific Islander, Native Hawaiian or other pacific islander, more than one, unknown, refused, and missing

Other** includes more than one, unknown, refused, and missing

Other*** includes missing, refused, and unknown PLwCP = Persons living with chronic pain

Labels for levels of chronic pain impact

### Labels for levels of chronic pain impact

PLwCP participants were invited to share the words they used to describe different levels of chronic pain impact. Participants quickly generated descriptors for pain severity but had difficulty coming up with general labels for graded levels of pain impact. The facilitator prompted participants, telling them that, in other studies, labels like “none/mild/moderate/severe” were used. She also put forth the labels used for the GCPS-R (i.e., “mild/bothersome/high” chronic pain impact). Participants immediately expressed support for such labels but energetically disagreed about using “moderate” vs. “bothersome.” Negative critiques of “moderate” included the opinion that “mild” and “moderate” were too similar and, therefore, hard to distinguish. Some thought “bothersome” was a better category to distinguish between “mild” and “high.” Negative critiques of “bothersome” included the opinion that the word implies an emotional component and “doesn’t fit” with “mild and moderate.” Consensus was not reached, so the facilitator asked the participants to vote. The results were 5–4 for “none/mild/moderate/high” over “none/mild/bothersome/high”; one abstained.

### Threshold placements

#### Bookmarking results

**Fig. 1:**
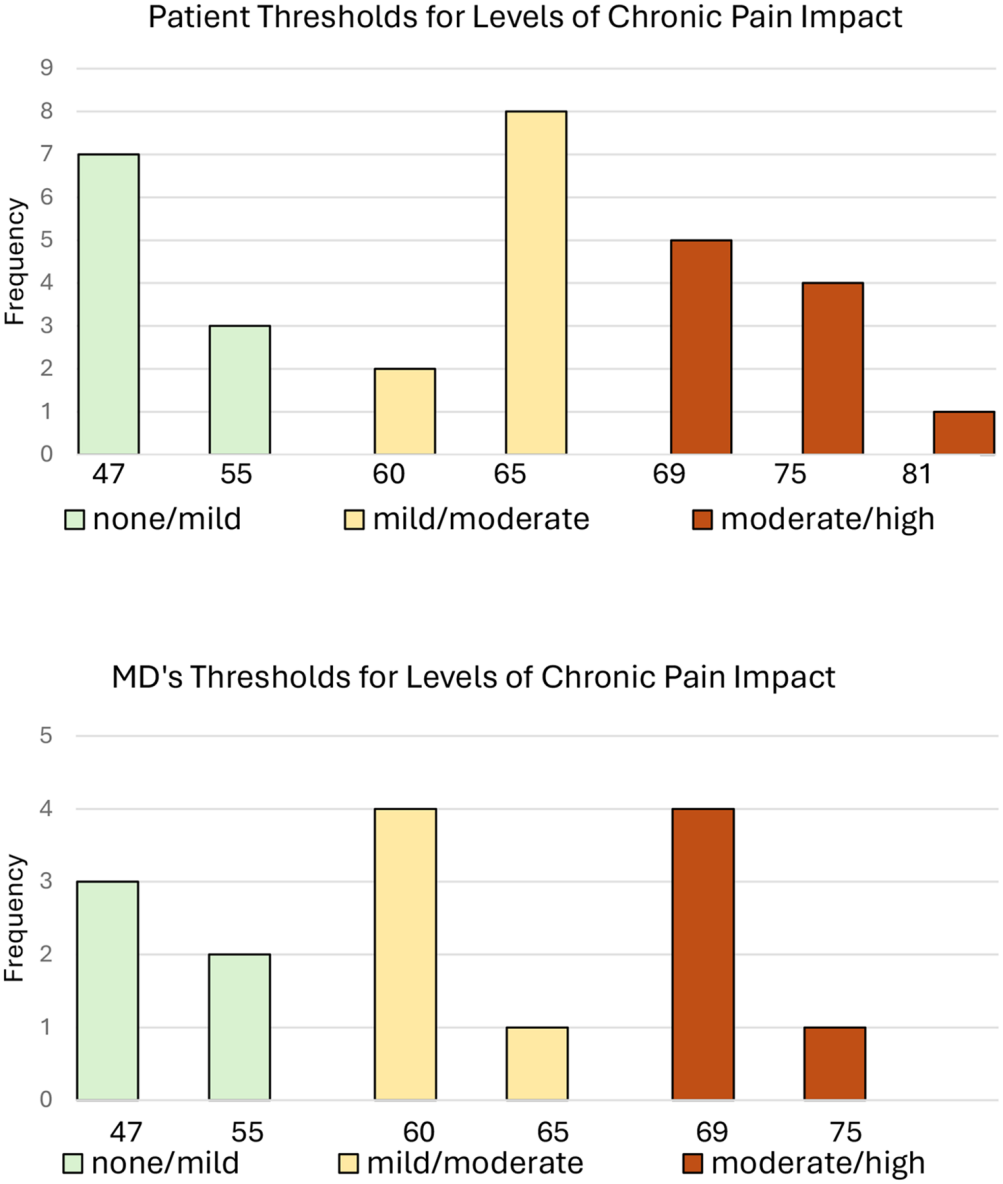
Individual panel members’ threshold locations based on PROMIS pain interference scores

Figure 1 represents the distributions of individual threshold scores for the PLwCP and Physician panels. Neither the PLwCP nor the Pain Clinicians reached unanimity. Most participants were within one vignette of each other (5 points on the T-score metric) in their placement of bookmarks. The exception was one PLwCP whose placed the bookmarker for high chronic pain impact 2 vignettes (12 points on the T-score metric) higher than the modal placement. Four PLwCP and no physicians changed their original threshold locations.

**Fig. 2:**
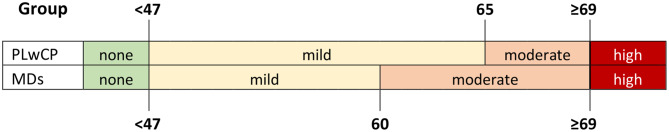
Comparison of Threshold Locations Based on PROMIS Pain Interference Scores and the Bookmarking Results for Patients Living with Pain (PLwCP) and Pain Clinicians. Consequential Validity

Figure 2 compares the thresholds for the clinicians and the PLwCP panel. There was substantial agreement between the two panels. What distinguished the panels was the threshold for moderate chronic pain impact. Clinicians set this threshold as one vignette lower (5 T-score points) than the panel of PLwCP.

**Fig. 3:**
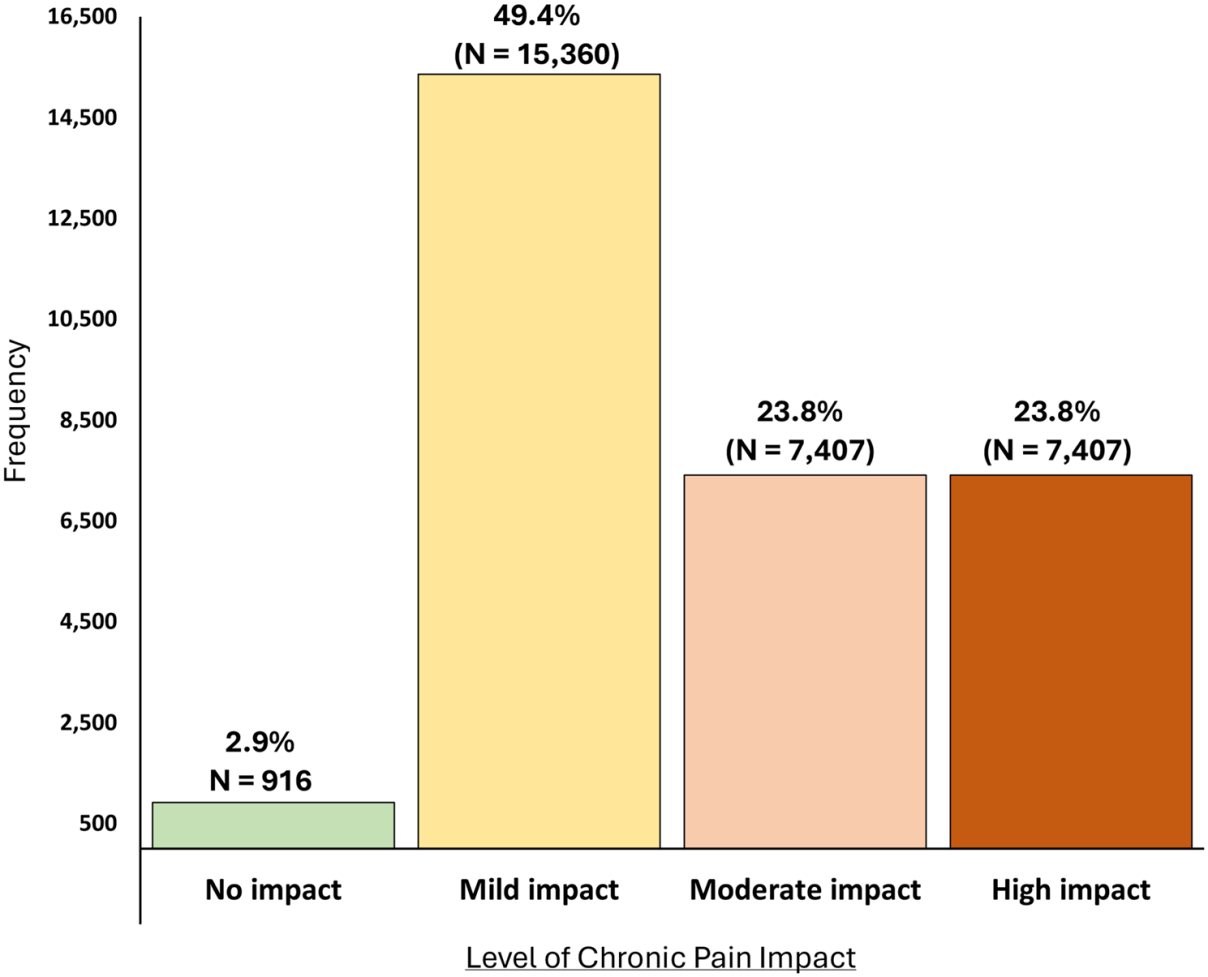
Distributions by Level of Chronic Pain Impact Based on Patient Panel Bookmarking Results (N = 31,090)

We applied the modal threshold values obtained from the panel of PLwCP to the Learning Health Care Sample. Table 1 describes the demographics for this sample (N = 31,090). Figure 3 shows the distribution of Pain Impact levels in the comparison sample drawn from CHOIR. The levels were defined based on the modal thresholds obtained from the Panel of PLwCP. The distribution by level of chronic pain impact was consistent with expectations for patients being seen in a tertiary pain management center. Only a small percentage of the sample were classified as having no impact (2.9%). A substantial proportion of participants were classified as having HICP (23.8%).

The demographic characteristics of the Social Determinants of Health Study Sample (n = 409) are reported in Table 1. Table 2 compares levels of chronic pain based on two scoring rubrics—the GCPS-R and the modal thresholds of the PLwCP Bookmarking Panel. Recall the label for the 3^rd^ level varied between the classification systems (PROMIS-PI—“moderate”; GCPS-R—“bothersome”). The overall Spearman Rho Correlation was 0.492 (p <.001). The modest correlation may have been due, in part, to the differences in labeling of the third level (“moderate” in the Bookmarking results, “bothersome” in the GCPS-R). Additionally, the PROMIS-PI items assess pain impact in the *past 7 days*, whereas the first two items of GCPS-R, which are used to determine no or high pain impact, assess its frequency and impact in the *past 3 months*. There was substantial variation in classification between the two rubrics, especially at the level of mild impact (PROMIS PI = 261; GCPS-R = 59) and high impact (PROMIS PI = 65; GCPS-R = 238).

**Table 2:** Classification of Levels of Chronic Pain based on the Revised Graded Chronic Pain Scale (GCPRS-R) and the Modal Bookmark Locations from the Panel of Persons Living with Chronic Pain (PLwCP)

		GCPS-R Classification				Total
		no impact	mild impact	*bothersome	high impact	
PLwCP Bookmark Classification	no impact	6	5	0	0	11
	mild impact	37	53	58	113	261
	*moderate impact	3	0	4	65	72
	high impact	0	1	4	60	65
Total		46	59	66	238	409

*Note that the label for the third level is different in the two scoring rubrics

#### Known-groups analysis

A one-way ANOVA was conducted to examine whether the PROMIS-PI T-scores differed significantly among groups with no, mild, moderate, and high impact chronic pain, as classified by the GCPS-R. The omnibus results indicated that PROMIS-PI T-scores were significantly different among the four groups, *F*(3, 405) = 96.188, *p* <.0001, η^2^ =.416. Table 3 presents the Bonferroni post-hoc test results. Pairwise comparisons revealed that PROMIS-PI T-scores were significantly different between all groups (*p*s <.0001) except between the no-impact and mild-impact groups

**Table 3:** Bonferroni post-hoc test for comparing the PROMIS-Pain Interference T scores between the levels of Chronic Pain based on the Revised Graded Chronic Pain Scale (GCPRS-R)

*M* and *SD* of PROMIS PI T scores							
No impact (n = 46)		Mild impact (n = 59)		Bothersome (n = 66)		High impact (n = 238)	
*M*	*SD*	*M*	*SD*	*M*	*SD*	*M*	*SD*
55.8	7.1	54.8	5.7	60.7	4.6	66.2	5.5
Group Comparison							
		Mean Difference		SE	95% CI		*p*
No vs Mild impact		0.91		1.10	−2.00	3.83	1.0000
No vs Bothersome		−4.96		1.07	−7.81	−2.11	<.0001
No vs High impact		−10.45		0.90	−12.84	−8.06	<.0001
Mild vs Bothersome		−5.88		1.00	−8.53	−3.22	<.0001
Mild vs High impact		−11.37		0.81	−13.53	−9.21	<.0001
Bothersome vs High		−5.49		0.78	−7.56	−3.43	<.0001

## Discussion

Our study is the first to define levels of chronic pain impact using input directly from PLwCP and pain clinicians. We found that PRO-Bookmarking participants could distinguish among pain impact levels based on “score stories” that evoked what it was like to live with graduated levels of pain impact. We successfully established threshold scores for PROMIS-PI that delineate none, mild, moderate, and high chronic pain impact by employing the PRO-Bookmarking method. These thresholds facilitate a nuanced understanding of pain’s influence on daily functioning, allowing for more precise clinical assessments and interventions.

Substantial prior work has been completed on classifying pain intensity from 0–10 pain ratings [15–19]. Less attention has been paid to the classification of levels of pain impact, also known as pain interference. The GCPS-R is a notable exception. We identified two additional studies that estimated associated verbal descriptors of pain interference levels with scores on a PRO [20, 21]. Both of these used PRO Bookmarking method to bookmark the PROMIS-PI measure, but neither attempted to define ranges for HICP.

Our results were based on both clinical opinion and the “patient’s voice.” The importance of including the patient’s experience opinions in developing a measure and interpreting its scores has become axiomatic. A strength of our study is that we accomplished that using a novel method. The results of our study, however, demonstrate that patients are not a homogenous group, and their voices can vary. This was particularly evident in the Bookmarking Panel of PLwCP’s discussions on how to name levels of chronic pain impact. A consensus could not be reached on using “moderate” versus “bothersome” for the 3^rd^ level of impact. It was eventually settled by a 5–4 vote, with one abstaining.

There was no consensus on bookmark placement in the panel of clinicians nor in the panels of PLwCP with regard to the placement of bookmarks. Though patients agreed substantially in thresholds for the first two levels (i.e., none/mild impact, mild impact/moderate impact), there was less agreement on the threshold that separated “moderate impact” and “high impact” chronic pain.

In comparing the results from the physician panel and from PLwCP, we noted that PLwCP required greater impact for defining HICP. A number of previous PRO measure Bookmarking studies have found similar results [20, 22–25], though this has not been found to be true for all measures or for all studies [21]. A potential explanation for this tendency, where it exists, is the ability of people to accommodate to their condition [23].

The results of the study supported the consequential validity of the threshold values. Based on the modal threshold values from the PLwCP Panel, a large proportion of patients receiving care at a tertiary pain management center would be classified as having HICP and very few as having no pain impact. Admittedly, this is quite a low bar for establishing consequential validity. Its chief value is as a referent for comparing future chronic pain impact classifications.

The comparisons of PLwCP classifications with those of the GCPS-R supported the consequential validity of the former. We expected to find similarities in classifications, and we did. The modesty of the level of agreement is of interest. One contributing factor could be the difference in the naming of one of the levels (i.e., moderate impact vs. bothersomeness). The fact that the GCPS-R rubric classified more patients as having HICP compared to the results from the panel of PLwCP is notable and consistent with the aforementioned findings that patients may place higher thresholds than clinicians [20, 22–25]. There are consequences in using one system over the other when identifying HICP, but we cannot say that one under- or over-represents the prevalence of HICP since the operational definition of HICP is still evolving. The merits of using one or both classification systems may become clearer as they are applied in more samples.

We acknowledge the limitations of threshold-based classifications of levels of outcomes. We expect the thresholds we derived will be useful for describing and interpreting levels of pain impact in samples of PLwCP and for conducting epidemiological studies and predictive modeling. We do not expect the thresholds to be as useful at the clinical level. Collapsing continuous data into four meaningfully labeled categories improves interpretability but concurs substantial loss of fine-grained information. A patient’s classification as having HICP can give a clinician a broad understanding of what the patient is experiencing, but the information is too broad, for example, to inform treatment decisions or evaluate improvement.

We also acknowledge limitations at the current study, including the fact that the facilitator prompted participants to discuss label names. This decision was prompted by the time constraints of the scheduled meeting and the lack of participant response. This choice, though perhaps defensible logistically, was far from ideal. By prompting participant using examples with 5 response categories, she likely biased participants to limit their discussion to a “none” category and 4 additional levels. Future research could dedicate one or more focus groups to eliciting the perceptions of PLwCP with regard to the number and levels of chronic pain impact. However, this study limitation does not nullify the study findings. The inclusion of “high chronic pain impact” aligned with operationalizing, from the perspective of patients, what constitutes what researchers have labeled HICP. Also, the labels that were used align closely with the GCPS-R labels.

Another limitation is that a single panel was used for each group, one for PLwCP and one for clinicians. In a recent study [25], Rothrock, et al compared the results of four Bookmarking studies using the same protocol with PLwCP. They found substantial agreement among individual study recommendations but also found variations. They concluded that best practice for Bookmarking would include more than a single study. Recently a Center for Excellence in Regulatory Science and Innovation (CERSI) Project [26] developed Beta software to support digital Bookmarking. As the software is further developed, it will support Bookmarking with much larger samples and lower costs compared to in-person Bookmarking.

Additionally, we note that our sample was drawn from a tertiary pain clinic and the panel of PLwCP had mean PROMIS-PI T scores of 66.6 (SD = 6.5) for the bookmarking group, well above the population mean of 50.0 (SD = 10.0). Future studies should explore the impact of panel members’ levels of pain interference with the location of their bookmarks. This could not be evaluated in the current study both because of the homogeneity of self-reported pain and the small sample size. Future research should consider employing multiple panels and using community samples to enhance the findings’ generalizability and potentially refine the threshold scores. Additionally, the decision to anchor vignettes 0.5 standard deviations apart was pragmatic but may have influenced the specific thresholds identified. Exploring different anchoring intervals could provide further insights into optimal scoring thresholds for pain impact classification.

In conclusion, the PRO-Bookmarking method offers a viable approach for deriving clinically meaningful thresholds for classifying levels of chronic pain impact. The PROMIS-PI rubric has two advantages compared to that of the GCPS-R. First, the rubric benefited from the input of people living with the pain impact being classified. Second, the PROMIS-PI rubric can be applied post hoc to any sample that included responses to the PROMIS-PI measure. However, replication studies are needed to confirm or revise the threshold values found in this study. Additionally, more qualitative work is needed to define the meaning of chronic pain from the perspectives of different stakeholders.

While further work is needed, our findings from this study make a significant contribution to the field of pain measurement and management. Through continued collaboration among researchers, clinicians, and patients, we can ensure that our approaches to measuring and managing chronic pain are both scientifically robust and deeply human-centered.

## Electronic supplementary material

Below is the link to the electronic supplementary material.

Supplementary Material 1
