# Supplementary material for: Defining chronic pain impact levels: a patient-clinician approach using PROMIS® pain interference scores
Source: J Patient Rep Outcomes. 2025 Aug 18;9:103. doi: 10.1186/s41687-025-00908-y (PMC12361012; doi:10.1186/s41687-025-00908-y)
Supplement: Supplementary file 1 — Supplementary Material 1 [file 41687_2025_908_MOESM1_ESM.docx]

**Ms. You’s Pain Interference (41)**

This is how Ms. You described his pain *over the last 7 days*:

- Her pain never interfered with the things she usually does for fun.
- Her pain never prevented her from sitting for more than 10 minutes.
- Her pain interfered not at all with her ability to do household chores.
- Her pain interfered not at all with her enjoyment of life.
- Her pain did not at all interfere with work around the home.
- Her pain never made her feel depressed.

**Mr. Thomas’ Pain Interference (53)**

This is how Mr. Thomas described his pain *over the last 7 days*:

- His pain interfered a little bit with his day to day activities.
- Pain interfered a little bit with his enjoyment of recreational activities.
- Never was his pain so severe he could think of nothing else.
- His pain never interfered with his close personal relationships.
- His pain never interfered with doing his tasks away from home (e.g. getting groceries running errands).
- His pain interfered a little bit with his household chores.

**Mr. Hayes’ Pain Interference (57)**

This is how Mr. Hayes described his pain *over the last 7 days*:

- His pain sometimes made him feel discouraged.
- Rarely, he avoided social activities because they might make him hurt more.
- Pain interfered a little bit with his ability to concentrate.
- His pain sometimes prevented him from walking more than 1 mile.
- His pain made it a little bit difficult to fall asleep.
- His pain never prevented him from standing for more than 30 minutes.

**Ms. Lewis’ Pain Interference (63)**

This is how Ms. Lewis described her pain *over the last 7 days*:

- Her pain interfered somewhat with her enjoyment of social activities.
- Her pain was sometimes so severe that she could think of nothing else.
- Her pain interfered a little bit with her ability to remember things.
- Pain interfered somewhat with her enjoyment of recreational activities.
- Sometimes, pain made it hard to plan social activities because she didn't know if she would be in pain.
- Pain interfered very much with her enjoyment of life.

**Ms. Stewart’s Pain Interference (66)**

This is how Ms. Stewart described her pain *over the last 7 days*:

- Quite a bit, her pain prevented her from sitting for more than 10 minutes.
- Her pain sometimes made her feel depressed.
- Her pain often prevented her from walking more than 1 mile.
- Pain was often distressing to her.
- Pain interfered quite a bit with work around the home.
- Pain often restricted her social life to her home.

**Mr. Evans’ Pain Interference (71)**

This is how Mr. Evans described his pain *over the last 7 days*:

- His pain interfered quite a bit with his day to day activities.
- Pain interfered quite a bit with his ability to remember things.
- His pain interfered very much with the things he usually does for fun.
- His pain always made it difficult to plan social activities.
- Pain interfered quite a bit with his relationships with other people.
- Pain interfere very much with his ability to work (including work at home).

**Ms. Coleman’s Pain Interference (81)**

This is how Ms. Coleman described her pain *over the last 7 days*:

- Pain always restricted her social life to her home.
- Pain interfered very much with her enjoyment of life.
- Her pain always prevented her from sitting more than 10 minutes.
- Her pain felt very much like a burden to her.
- Pain always kept her from socializing with others.
- Pain always made her feel anxious.
